# The Interrelationships of Coping Styles and Professional Burnout Among Physiotherapists: A Cross-Sectional Study: Erratum

**DOI:** 10.1097/01.md.0000469916.53101.05

**Published:** 2015-07-17

In the article “The Interrelationships of Coping Styles and Professional Burnout Among Physiotherapists: A Cross-Sectional Study”,^1^ which appeared in Volume 94, Issue 24 of *Medicine*, the term TOC (task-oriented coping) was incorrectly spelled as TOP in 3 locations in the text. The article has since been corrected online.
